# Trends in all-cause mortality and leading causes of death from 2009 to 2019 among older adults in China

**DOI:** 10.1186/s12877-023-04346-7

**Published:** 2023-10-11

**Authors:** Jian Wu, Zihan Mu, Shuai Jiang, Yudong Miao, Yanyu Tang, Jing Wang, Suxian Wang, Yaojun Zhao

**Affiliations:** 1 Department of Health Management, College of Public Health, Zhengzhou University, Henan, People’s Republic of China; 2 Operation Management Department, Fuwai Hospital, Central China Fuwai Hospital of Zhengzhou University, Zhengzhou, Henan 450001 People’s Republic of China; 3 The First Affiliated Hospital of Zhengzhou University, Henan, People’s Republic of China

**Keywords:** Mortality trends, Leading causes of death, Older adults, China

## Abstract

**Background:**

This study aimed to determine long-term variations in mortality trends and identify the leading causes of death among older adults in China from 2009 to 2019 so as to propose interventions to further stabilise the mortality rate among older adults and facilitate healthy ageing.

**Methods:**

We extracted data from the China Death Surveillance database from 2009 to 2019 for all-cause mortality and cause-specific death among individuals aged ≥ 65 years. A joinpoint regression model was used to estimate mortality trends by calculating the annual percentage change (APC). A trend chi-square test was used to estimate sex differences in mortality, and descriptive analysis was used to estimate the leading causes of death. Semi-structured expert interviews were conducted to examine health interventions for older adults.

**Results:**

We observed an overall declining trend in age-adjusted mortality rates among older adults aged ≥ 65 years in China from 2009 to 2019 (APC, -2.44; *P* < 0.05). In this population, the male mortality rate was higher than the female mortality rate during this period (*P* < 0.05). However, the mortality rate among older adults aged ≥ 85 years increased since 2014, particularly among females. Cardiovascular disease (CVD) was the leading cause of death among older adults aged 65–84 years, whereas ischaemic heart disease was the leading cause of death among individuals aged ≥ 85 years, especially among females. The majority of injuries resulting in death were caused by falls, showing an increasing trend.

**Conclusions:**

CVD is a major cause of death among older adults aged ≥ 65 years in China, and relevant health intervention strategies should be implemented from the perspectives of physiology, psychology, and living environment. The change in the mortality trend and the distribution of cause of death among older adults aged ≥ 85 years is noteworthy; a diagnostic and management model centred around females aged ≥ 85 years should be implemented. Additionally, a multidimensional fall prevention strategy involving primary medical institutions and care services needs to be implemented to reduce the risk of falls among older adults.

**Supplementary Information:**

The online version contains supplementary material available at 10.1186/s12877-023-04346-7.

## Background

China has the largest older adult population worldwide (1, 2). China’s seventh national census in 2020 reported that the population aged ≥ 65 years had reached 190 million people. According to the World Health Organization, if ≥ 7% of the total population is aged ≥ 65 years in a country or region, it indicates the emergence of an ageing society. Currently, the proportion of people aged ≥ 65 years in China is 13.5%. Compared with the sixth national census in 2010, the proportion of people aged ≥ 65 years increased by 4.63% points. The 2021 Strategy Research Report on Actively Coping with Population Aging noted that ‘the degree of population ageing in China continues to increase, and the speed of population ageing has accelerated significantly’. Owing to China’s increasing ageing population, medical care costs for older adults have resulted in significant individual and societal health and economic burdens. In 2021, guidelines were published in China concerning measures to support the implementation of a national strategy to address population ageing; promote its efforts to address population ageing in the most recent context; and boost the sense of fulfilment, happiness, and safety among older adults to achieve healthy ageing.

Mortality rates and leading causes of death among older adults are important public health monitoring indicators [3]. In addition to reflecting changes in older adult mortality, trend-related analyses can provide important information for monitoring public health. Clarification of the main causes of death among older adults can serve as a means of determining whether these causes have changed and of providing guidance for health departments in evaluating and adapting public health policies. It can also serve as an evidence-based basis for developing targeted health management strategies. Furthermore, identifying the main causes of death in older adults can provide a basis for further research that will examine how these causes of death are changing.

Globally, most mortality rate and cause of death studies involving older adults have focused on mortality trends, influencing factors, and models. A previous study found that the proportion of in-hospital deaths showed a decreasing trend, with deaths from pneumonia accounting for the highest proportion of in-hospital deaths [4]. Previous studies among older adults have reported that physical activity, cognitive leisure activity, and adequate sleep were associated with lower mortality rates, whereas a low socioeconomic status was associated with increased mortality rates [5–8]. One study reported that cancer mortality rates among older adults showed a decreasing trend in multiple countries worldwide, with relatively high cancer mortality rates observed in central and eastern European countries [9]. According to a study in Brazil, fall-related mortality rates among older adults are declining [10].

In 2021, a prediction model was constructed in another study to determine in-hospital mortality rates for patients aged ≥ 75 years with pneumonia, aiming to improve their health management levels [11]. However, relevant studies are limited, and little is known about long-term variation trends in mortality rates and leading causes of death among older adults in China. Therefore, this study aimed to determine the mortality trend of older adults aged ≥ 65 years in China from 2009 to 2019 and to reflect changes in the mortality of older adults through trend analysis. Based on the analysis of mortality trends, we further analysed the leading causes of death among older adults aged ≥ 65 years in China from 2009 to 2019 and obtained specific health intervention suggestions by conducting expert interviews, so as to provide targeted health intervention strategies to further stabilise the mortality rates of older adults.

## Methods

### Data sources

**Fig. 1:**
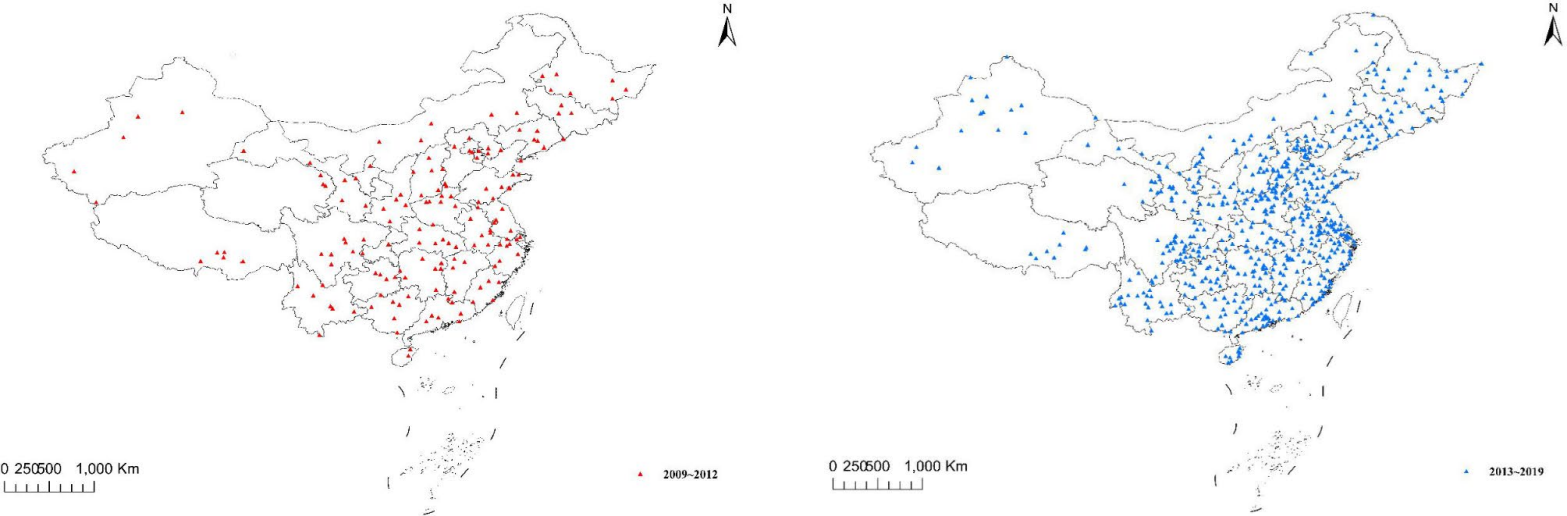
A map of the DSP across different years. DSP, disease surveillance points

The study was based on the China Death Surveillance Dataset (CDS) from 2009 to 2019, which was collected by the National Disease Surveillance Points System (DSP). The DSP uses multi-stage stratified cluster random sampling to select monitoring sites in 31 provinces, with a total of 161 monitoring sites selected from 2009 to 2012, involving a population of 77 million and accounting for 6% of the national population. Since 2013, the former Ministry of Death Statistics System, DSP system, and other death reporting systems were integrated; as a result of this integration, the number of monitoring points has expanded to 605, covering more than 300 million people, accounting for 24% of the country’s population. Details of the monitoring point are shown in Fig. 1. Furthermore, the process for selecting a monitoring site is shown in Appendix 1.

### Data collection and quality

#### Registration of deaths

The DSP is designed to register all deaths that occur within each jurisdiction, including deaths among registered and non-registered Chinese residents. All medical and health institutions at various levels are required to supply relevant information in a death information report. From 2009 to 2019, the average percentage of ill-defined causes was 1.33%; the highest percentage was 1.59% and the lowest was 1.19%. The death registration process is shown in Appendix 2.

#### Death data entry and reporting

All deaths in the DSP are reported online through the cause-of-death registration and report information system of the Chinese Center for Disease Control and Prevention, which reviews data reported from the provinces and verifies and revises the data as needed.

#### Data quality control

Omission is unavoidable; therefore, DSP sets the exclusion criteria for monitoring points, and data from monitoring points that are seriously underreported and potentially affect the overall outcome are eliminated. The exclusion criterion of the DSP system from 2009 to 2012 is that the mortality rate of monitoring points is < 4.5%, and the exclusion criterion of newly added monitoring points since 2013 is that the mortality rate is < 5%.

### Quantitative analysis

Data from the CDS for fields such as sex, age, and death status were selected. Mortality and cause of death among older adults aged ≥ 65 years were used as the main analysis indicator. The joinpoint regression model was used to estimate the age-adjusted mortality (AADR) trend in older adults aged ≥ 65 years by calculating the annual percentage change (APC). The principle of the joinpoint model is to establish all possible join points and calculate the corresponding error sum of squares and mean square error in each possible case; to select the points with the lowest mean square error as join point; and to fit the equation parameters according to the selected join points and interval function. The joinpoint models include the 0 joinpoint model (reflecting the trend of change throughout the study period) and multi-joinpoint model (reflecting the trend of change in each period). The analysis starts with 0 join points and tests and evaluates whether one or more join points need to be entered into the model again to compare the model to the best-fit data.

The AADR is a weighted average of mortality rates, where the mortality rates are calculated for different age groups and the weights are the proportions of persons in the corresponding age groups of a standard population. The AADR for an age group comprising the ages *x* through *y* is calculated using the following formula:

$${AADR}_{x-y}=\sum _{i=x}^{y}\left[\left(\frac{{count}_{i}}{{pop}_{i}}\right)\times \text{100,000}\times \left(\frac{{stdpop}_{i}}{{\sum }_{i=x}^{y}{stdpop}_{i}}\right)\right]$$

Count represents the number of cases in the *i*^th^ age group, pop_*i*_ is the relevant population for the same age group, and stdpop_*i*_ is the standard population for the same age group.

The APC helps characterise trends of mortality and prevalence over time. When the Log Transformation option on the Input File tab is *ln(y) = xb*, then the output calculates the estimated APC. Rates that change at a constant percentage annually show a linear change on a log scale. Therefore, to estimate the APC for a series of data, the following regression model is used:

$log\left({R}_{y}\right)={b}_{0}+{b}_{1}y$ where $log\left({R}_{y}\right)$ is the natural log of rate in year $y$.

The APC from year $y$ to year$y+1=\left[\frac{{R}_{y+1}-{R}_{y}}{{R}_{y}}\right]\times 100$

$$=\frac{\left\{{e}^{{b}_{0}+{b}_{1}\left(y+1\right)}-{e}^{{b}_{0}+{b}_{1}\left(y\right)}\right\}}{{e}^{{b}_{0}+{b}_{1}\left(y\right)}}\times 100$$

$$=\left({e}^{{b}_{1}}-1\right)\times 100$$

A trend chi-square test was used to estimate sex differences in mortality. A descriptive analysis was used to estimate the causes of death among people aged ≥ 65 years in China from 2009 to 2019. The disease classification used in this study was the 10th revision of the International Classification of Diseases.

### Qualitative analysis

Semi-structured expert interviews were conducted on health interventions for those aged ≥ 65 years. The interviews were conducted face-to-face or online and were analysed using Colaizzi’s seven-step method. This method focuses on participants’ experiences and feelings to identify common patterns rather than individual characteristics among the subjects. The following scientific approach ensures the authenticity of the experiences gathered by participants: (1) read the information carefully and repeatedly; (2) extract important statements relevant to the purpose of the study; (3) code recurring views; (4) pool coded and meaningful perspectives into thematic clusters; (5) comprehensively describe the thematic cluster; (6) summarise similar views to form the final theme; (7) feed the results back to the study participants for validation, with interviewers and analysts repeatedly comparing and calibrating the data analysis results to ensure accuracy.

#### Participants

The selection criteria for participants were as follows: (a) working in fields such as public health, epidemiology, or gerontology; (b) having > 5 years of service; and (c) volunteering for this interview. We contacted 15 potential interviewees to participate in the interview, of whom 14 agreed to participate (participation rate, 93.33%). Five respondents were employed in government health departments (35.71%), four in hospitals (28.57%), three in universities (21.43%), and two in medical companies (14.29%). Among the 14 interviews conducted, eight were face-to-face interviews and six were conducted online.

Each interview lasted no less than 40 min and was conducted by two interviewers (the interviewer and recorder). In addition to possessing a PhD in epidemiology and public health, the interviewer had experience in conducting qualitative interviews and had worked for 15 years in government health departments. Thus, the interviewer was qualified to carry out this research.

#### Interview outline

In order to determine the interview outline, we consulted relevant literature, sought the opinions of experts, and selected two experts for each interview (see Appendix 3 for details on the interview outline).

### Statistical analysis

Joinpoint Regression Program version 4.9.1.0 software was used for the joinpoint regression model and SPSS version 26.0 software was used for the trend chi-square test. Within 24 h of each interview, the recording was analysed using Colaizzi’s seven-step method. Two researchers independently reviewed the interview materials, summarised and extracted meaningful statements, and formulated the themes present. Differences were considered statistically significant at *P*-values < 0.05.

## Results

### Mortality trends in China from 2009 to 2019 among older adults aged ≥ 65 years

**Fig. 2:**
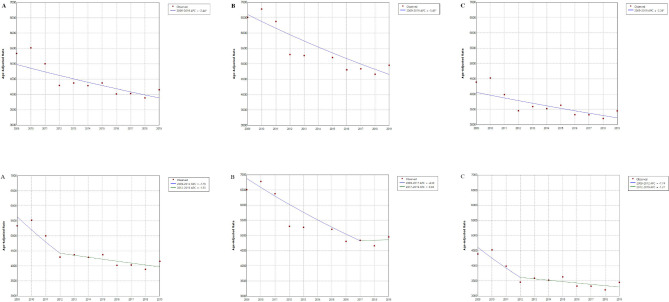
Mortality trends among older adults aged ≥ 65 years in China (2009–2019). **A**, total; **B**, male; **C**, female. *P < 0.05

Crude mortality rates in China from 2009 to 2019 among older adults aged ≥ 65 years are shown in Table 1. The joinpoint regression model showed that age-adjusted mortality rates for individuals aged ≥ 65 years in China showed a declining trend (APC, -2.44, *P* < 0.05). Among older adults of different sexes, the declining trend in age-adjusted mortality rates in males (APC, -3.43, *P* < 0.05) was greater than that in females (APC, -2.26, *P <* 0.05). The multi-joinpoint model demonstrated that the mortality rate trends of males and females aged ≥ 65 years had inflection points in 2017 (2009–2017 and 2017–2019) and 2012 (2009–2012 and 2012–2019), respectively. However, no statistical significance was noted in the declining trend of each period (APC, -4.33, *P =* 0.131; APC, 0.38, *P =* 0.980; APC, -7.74, *P =* 0.202; APC, -1.31, *P =* 0.117; Fig. 2).

**Table 1:** Mortality rates among older adults aged ≥ 65 years in China (2009–2019) **(1/100,000)**

Years	65–69 years	70–74 years	75–79 years	80–84 years	≥ 85 years
2009	1729.36	3062.05	5211.17	9335.23	28062.25
2010	1668.99	2909.60	5003.18	9518.91	32266.63
2011	1542.62	2628.43	4629.82	8695.81	28651.26
2012	1654.14	2756.70	4726.38	8363.86	16753.50
2013	1717.01	2848.76	4746.19	8452.53	17021.60
2014	1755.17	2819.95	4637.08	8051.63	16639.69
2015	1798.06	2841.84	4578.80	8118.88	17751.38
2016	1657.44	2522.77	4025.08	7482.19	17169.10
2017	1707.67	2532.16	3912.09	7443.60	17500.18
2018	1667.30	2423.87	3632.20	7035.65	17502.48
2019	1400.28	2500.49	4425.04	7336.01	19792.06

### Mortality trends according to sex and age among older adults aged ≥ 65 years in China

#### Trends throughout the period (2009–2019)

**Fig. 3:**
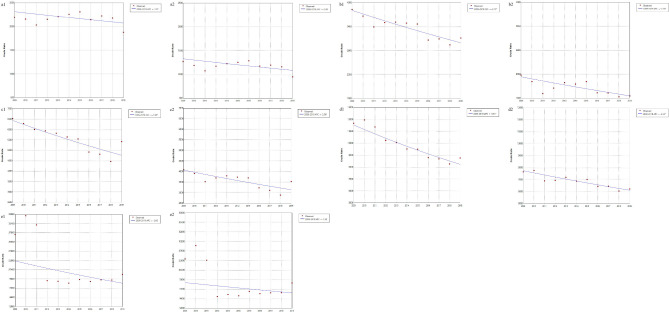
Mortality trends according to age and sex among older adults in China (2009–2019). **a1**, Males aged 65–69 years; a2, Females aged 65–69 years; trend *χ*^*2*^ = 3.885, *P <* 0.05; **b1**, Males aged 70–74 years; b2, Females aged 70–74 years; trend *χ*^*2*^ = 0.973, *P* = 0.324;**c1**, Males aged 75–79 years; c2, Females aged 75–79 years; trend *χ*^*2*^ = 12.466, *P <* 0.001; **d1**, Males aged 80–84 years; d2, Females aged 80–84 years; trend *χ*^*2*^ = 107.56, *P <* 0.001; **e1**, Males aged ≥ 85 years; e2, Females aged ≥ 85 years; trend *χ*^*2*^ = 254.063, *P <* 0.001. *P < 0.05

The mortality trends according to age and sex among older adults aged ≥ 65 years in China from 2009 to 2019 are shown in Fig. 3. In terms of trends throughout the period, the mortality rate of males and females aged 70–74 years (APC, -2.14, *P* < 0.001; APC, -2.10, *P* = 0.003), 75–79 years (APC, -2.96, *P =* 0.005; APC, -2.56, *P =* 0.005), and 80–84 years (APC, -3.31, *P* < 0.001; APC, -2.33, *P* < 0.001) showed a declining trend.

The trend chi-square test results showed that male mortality rates were higher than female mortality rates. The average mortality rates for males in each age group were 1.84 (65–69 years), 1.56 (75–79 years), 1.44 (80–84 years), and 1.23 (≥ 85 years) times greater than those for females.

#### Trends of different periods

**Fig. 4:**
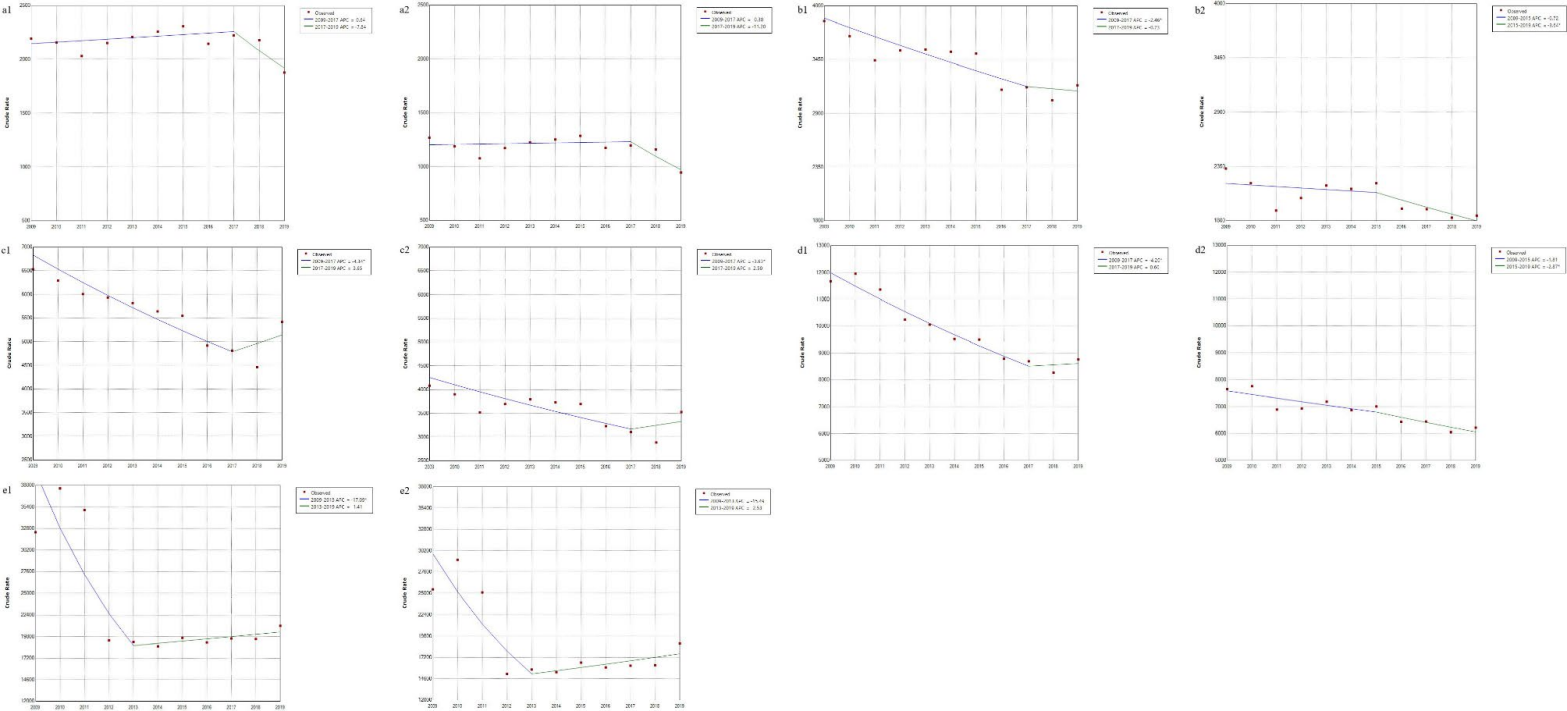
Mortality trends according to age and sex among older adults in China at different periods. **a1**, Males aged 65–69 years; a2, Females aged 65–69 years; **b1**, Males aged 70–74 years; **b2**, Females aged 70–74 years; **c1**, Males aged 75–79 years; **c2**, Females aged 75–79 years; **d1**, Males aged 80–84 years; d2, Females aged 80–84 years; **e1**, Males aged ≥ 85 years; **e2**, Females aged ≥ 85 years. *P < 0.05

The mortality trends of the different periods according to age and sex among older adults in China are shown in Fig. 4. The mortality rate trends of 70–74-year-old males and females showed an inflection point in 2017 (2009–2017; 2017–2019) and 2015 (2009–2015; 2015–2019), respectively. The mortality rates of males aged 70–74 years from 2009 to 2017 and females aged 70–74 years from 2015 to 2019 showed a declining trend (APC, -2.46, *P* = 0.012; APC, -3.64, *P* = 0.048). The mortality trends for both males and females aged 75–79 years showed an inflection point in 2017, with a declining trend in mortality from 2009 to 2017 (APC, -4.34, *P* < 0.001; APC, -3.63, *P* = 0.008).

The mortality rate trends of 80–84-year-old males and females showed an inflection point in 2017 (2009–2017; 2017–2019) and 2015 (2009–2015; 2015–2019), respectively. The mortality rates for males aged 80–84 years from 2009 to 2017 and of those for females in the same age range from 2015 to 2019 showed a declining trend (APC, -4.20, *P* < 0.001; APC, -2.87, *P* = 0.031).

The trend of mortality rates for both males and females aged ≥ 85 years showed an inflection point in 2013 (2009–2013 and 2013–2019). The mortality rate of males showed a decreasing trend (APC, -17.09, *P* = 0.043) from 2009 to 2013 and an increasing trend from 2013 to 2019 (APC, 1.41, *P =* 0.451); however, the increasing trend was not statistically significant. In terms of mortality trends for females aged ≥ 85 years, a declining trend was observed from 2009 to 2013 and an increasing trend was observed from 2013 to 2019; neither period showed a statistically significant trend (APC, -15.49, *P =* 0.063; APC, 2.53, *P =* 0.200).

### Leading causes of death among older adults aged ≥ 65 years in China

**Fig. 5:**
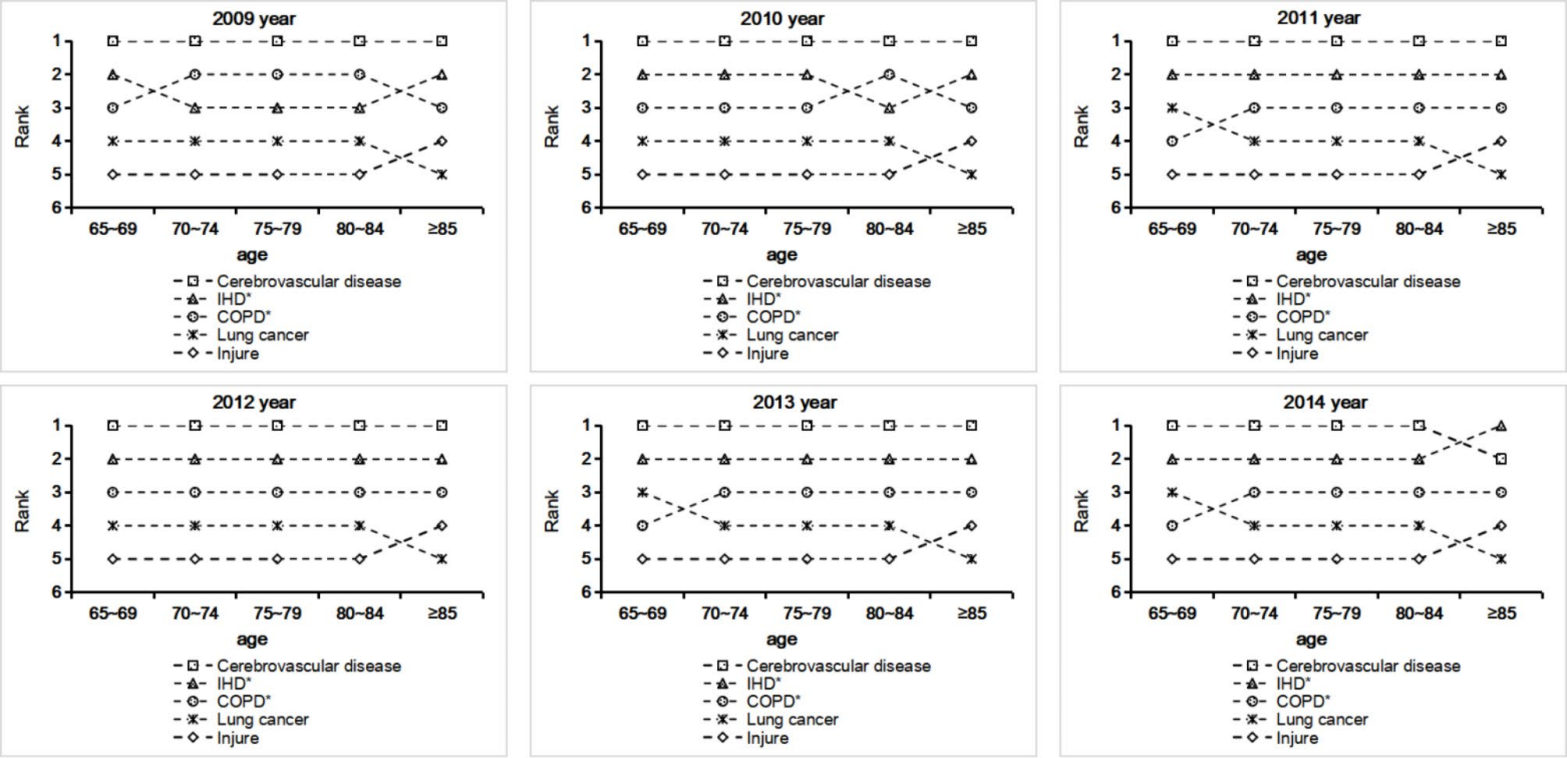
Cause of death rankings among older adults aged ≥ 65 years in China (2009–2019). COPD, chronic obstructive pulmonary disease; IHD, ischaemic heart disease. Note: From 2015 to 2019, the top five causes, and rank, of death of older adults aged ≥ 65 years in China were the same as those in 2014

The five leading causes of death among older adults aged ≥ 65 years in China from 2009 to 2019 were cerebrovascular disease (CVD), ischaemic heart disease (IHD), chronic obstructive pulmonary disease (COPD), lung cancer, and injury. CVD was the leading cause of death among those aged 65–84 years. However, among older adults aged ≥ 85 years, CVD was the leading cause of death from 2009 to 2013, while IHD was the main cause of death from 2014 to 2019 (Fig. 5).

According to the cross-analysis of deaths by age group and sex in this study, CVD was the leading cause of death in both males and females of all age groups from 2009 to 2013. As of 2014, the distribution of the leading causes of death has changed. Among females aged ≥ 85 years, IHD became the leading cause of death from 2014 to 2019. Among males aged ≥ 85 years, CVD was the leading causes of death in 2009, followed by COPD and IHD. However, from 2010 to 2016, deaths due to IHD surpassed those due to COPD. From 2017 to 2019, IHD was the leading cause of death, surpassing CVD (Appendix 4).

**Fig. 6:**
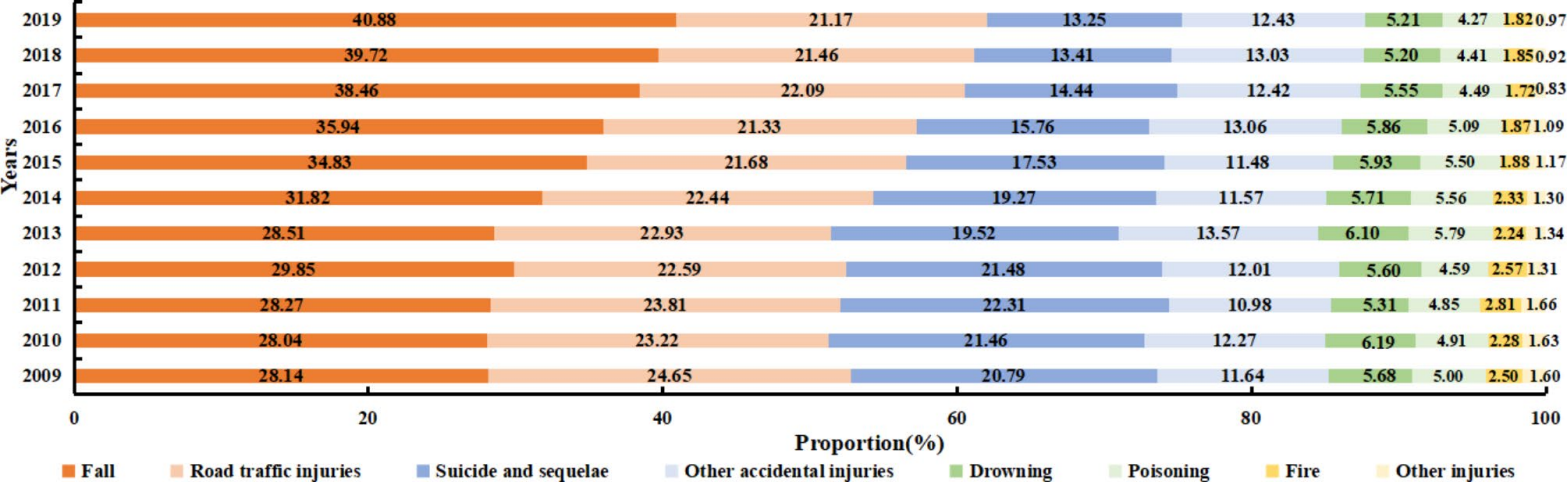
Causes of death due to injuries among older adults in China (2009–2019). Note: Other injuries include violence and war

Data concerning the proportion of specific injury mortality rates among older adults aged ≥ 65 years in China from 2009 to 2019 are summarised in Fig. 6. Suicide mortality rates showed a declining trend, while falls accounted for the highest proportion and showed an increasing trend, growing at a rate of 4.53% annually.

### Summary of expert interview

We analysed the interview data of experts and summarised three themes, which are shown in Table 2. In theme I, a total of 85.7% (12/14) of participants suggested that older male adults should be included in the priority group for health education, 71.4% (10/14) suggested that regular lectures should be conducted on the risks associated with unhealthy lifestyles, and all participants (14/14) stated that smoking and drinking should be reduced among older male adults.

**Table 2:** Themes identified through interviews with experts

Theme	Subtheme
I. Strengthening self-health management awareness among older males	i. Include older males in health education focus groups
	ii. Conduct regular education campaigns on the dangers of unhealthy lifestyles
	iii. Reduce the frequency of smoking and drinking among older males
II. Development of a programme for the integrated management of CVD and IHD in older adults	i. Conduct CVD and IHD prevention education regularly
	ii. Develop specific intervention programmes for CVD in older adults
	iii. Establish health records for high-risk groups with CVD and IHD
	iv. Improve the diagnosis and treatment of CVD
III. Strengthening falling-prevention intervention for older adults	i. Strengthen the focus on the harm of falls among older adults at a social level
	ii. Primary medical institutions should strengthen the prevention of falls among older adults
	iii. Improve the fall-prevention intervention technology for older adults at a social level

CVD, cardiovascular disease; IHD, ischaemic heart disease

In theme II, all participants (14/14) mentioned that education on the prevention of CVD and IHD should be strengthened, 64.3% (9/14) emphasised the importance of developing special intervention programmes for CVD in older adults, and 78.6% (11/14) of participants mentioned the need to establish health records for high-risk groups with CVD and IHD. Furthermore, 85.7% (12/14) of participants suggested improving the diagnosis and treatment of CVD as well as implementing a diagnostic and management model directed at females aged ≥ 85 years.

In theme III, 71.4% (10/14) of the participants mentioned that more attention should be paid to the adverse effects of falls among older adults, 92.9% (13/14) suggested that primary medical institutions should strengthen the prevention of falls among older adults, while 57.1% (8/14) suggested that fall intervention technology at the social level should be enhanced for older adults.

## Discussion

This study examined long-term variations in mortality trends and the leading causes of death among older adults aged ≥ 65 years in China from 2009 to 2019, aiming to facilitate improvements in quality of life and help promote targeted disease prevention strategies in this population.

Our study findings indicated a declining trend in all-cause mortality among older adults in China from 2009 to 2019, with male mortality rates generally being higher than female mortality rates. A study conducted in Liangshan Yi Autonomous Prefecture in China showed that the male mortality rate was approximately 1.47 times higher than the female mortality rate, and a study in the United States also reported higher male mortality rates [12, 13]. These findings are likely to be associated with different lifestyles among older adults. The proportion of older male adults who actively smoke cigarettes and consume alcohol is higher than that of female adults in China, and mortality rates among smokers/consumers of alcohol have been reported to be 3.14 times higher than those among non-smokers/non-consumers of alcohol [14, 15]. A healthy lifestyle can significantly reduce mortality and increase life expectancy [16].

Based on the findings of our study, we recommend that the Chinese government focus on reducing smoking and alcohol consumption among older male adults through conducting health education lectures, issuing health information leaflets, and using other education strategies to promote a healthy lifestyle.

This study found that the mortality rate of older adults aged ≥ 85 years has increased since 2014, especially among females. Although the joinpoint regression model suggests that the trend is not statistically significant, the increase is observable. We attempted to analyse changes in mortality trends in terms of the leading causes of death. Interestingly, the IHD-related mortality rate continued to increase among older adults aged ≥ 85 years, surpassing that of CVD and COPD. Furthermore, since 2014, IHD has been the leading cause of death, particularly among females aged ≥ 85 years.

IHD is a type of coronary artery disease characterised by diffuse fibrosis due to coronary artery atherosclerosis. The risk of IHD increases as physiological function in older adults deteriorates and blood flow to the heart gradually decreases. In fact, China’s mortality rate due to IHD has increased steadily since the 1990s, increasing by 20.6% in 2017 (17). Additionally, several studies have shown that females are more likely to die from IHD compared with their male counterparts (18). Pregnancy-related complications and ovarian disease increase female’s risk of developing IHD [19]. IHD represents a significant health burden due to its high morbidity and mortality rates. As the ageing population gradually increases in number, this health burden is likely to increase significantly [20–22].

Previous studies have indicated that obesity, high cholesterol levels, and diabetes mellitus are independent risk factors for IHD; in particular, high cholesterol levels, closely linked to diet, have been significantly associated with IHD [23, 24]. Based on the study interviews, we recommend that the Chinese government take measures to help optimise diet structure for older adults and focus on the prevention and treatment of IHD among older adults with obesity and diabetes mellitus. In addition, a diagnostic and management model centred around females aged ≥ 85 years should be implemented.

CVD was found to be the leading cause of death among older adults aged 65–84 years in China. Globally, CVD is not only the second leading cause of death but also the main cause of long-term severe disability and a serious public health burden [25, 26]. The identification of relevant risk factors plays a significant role in preventing CVDs [27–29]. Previous studies have found that hypertension, lack of exercise, excessive smoking and alcohol consumption, and a high-sodium diet are risk factors for CVD [30, 31]. Depression and cognitive impairment in older adults have also been independently associated with CVD [32]. Furthermore, exposure to atmospheric particulate pollutants has been shown to have significant adverse effects on the health of individuals with CVD [33].

Health interventions to address CVD need to be conducted among older adults, wherein relevant physiological, psychological, and environmental factors are considered. We recommend that official guidance be provided to encourage older adults to engage in physical activity and that relevant authorities provide allocated spaces in which to perform such physical activity. A healthy diet that promotes low sodium intake and appropriate intake of high-protein foods, such as fish, should be encouraged to help deter the onset of CVD among older adults [34]. Relevant authorities should focus on the mental health of older adults, and organising social activities to help them build good interpersonal relationships. Furthermore, there should be a focus on improving the living environment of older adults to reduce the risk of CVD due to environmental pollutants [35].

In this study, we found that falls accounted for the highest proportion of injuries leading to death among older adults in China, and that this proportion has been increasing over time. Our findings accord with those of previous studies and may possibly be explained by the increasing older adult population and life expectancy. Older adults are more likely to fall, as they age and deteriorate physically, and falls are more likely to result in fatalities in such older adults than in younger individuals [36, 37]. Falls are the second leading cause of accidental injury-related deaths worldwide, with one-third of older adults aged ≥ 65 years and 50% of older adults aged ≥ 80 years reporting falls each year [38]. Moreover, in the United States, annual fall-related healthcare costs among older adults have been reported to be approximately $50 billion [39]. In the context of an ageing population, prevention of falls should be a priority of global public health strategies for older adults [40, 41].

Falls are a complex health issue and risk factors among older adults of differing ages vary. Risk factors for falls among older adults aged < 70 years primarily involve environmental factors such as inappropriate footwear, poor lighting, and smooth floors [36], whereas risk factors for falls among older adults aged ≥ 80 years primarily involve physical factors such as multiple comorbidities, frailty, visual impairment, and loss of balance ability [42, 43]. In addition, a previous study reported that falls were more harmful to older adults who lived alone [44]. Our findings suggest that primary medical and health institutions should regularly screen for risk factors such as visual function and joint disease among older adults. Moreover, older adults require specialised fall prevention and care services, especially for individuals with multiple comorbidities and those living alone. The development and use of technical equipment for the prevention of falls and for protection among older adults should be accelerated to minimise fall-related injuries.

### Strengths and limitations

First, to the best of our knowledge, this is the first national study on mortality rates and the causes of death among the older adult population, including 31 provinces, in China. Second, China has been implementing medical reforms since 2009, and our study covers this critical period of transformation and development. Third, we examined mortality rates and the leading causes of death among older adults in the context of population ageing to help provide an evidence-based basis for achieving active ageing. Finally, we conducted semi-structured interviews with appropriately qualified and experienced individuals in clinical and public health fields to help ensure the robustness of our findings.

A limitation of our study was that factors such as geographical environment and economic development were not considered in relation to mortality rates and causes of death in different regions. Studies that consider these factors are needed to investigate mortality rates and causes of death throughout different regions of China. In addition, the mortality data in the CDS are all monitoring values, and there is no omission adjustment for mortality. Therefore, underreporting at monitoring points could be subsequently investigated to analyse adjusted mortality trends.

## Conclusions

CVD is a major cause of mortality among older adults aged ≥ 65 years in China, and health intervention strategies for CVD should be implemented from the perspectives of physiology, psychology, and living environment. The change in the mortality trend and distribution of cause of death among older adults aged ≥ 85 years is noteworthy; a diagnostic and management model centred around females aged ≥ 85 years should be implemented. Additionally, a multidimensional fall prevention strategy involving primary medical institutions and care services needs to be implemented to reduce the risk of falls among older adults.

### Electronic supplementary material

Below is the link to the electronic supplementary material.

Supplementary Material 1

Supplementary Material 2

Supplementary Material 3

Supplementary Material 4

## Data Availability

All data generated or analysed during this study are included in this published article.

## Abbreviations

CDS: China Death Surveillance Dataset
DSP: disease surveillance points
AADR: age-adjusted mortality
APC: annual percentage change
CVD: cardiovascular disease
IHD: ischaemic heart disease
COPD: chronic obstructive pulmonary disease
