# Supplementary material for: Trends in all-cause mortality and leading causes of death from 2009 to 2019 among older adults in China
Source: BMC Geriatr. 2023 Oct 11;23:645. doi: 10.1186/s12877-023-04346-7 (PMC10566094; doi:10.1186/s12877-023-04346-7)
Supplement: Supplementary file 1 — Supplementary Material 1 [file 12877_2023_4346_MOESM1_ESM.docx]

**Appendix 1**

The steps for selecting monitoring sites from 2009 to 2012 are as follows:

1. According to the classification method of the National Bureau of Statistics, all the districts and counties in China (mainland areas) are divided into three parts by region: east, central and west.

2. Define all counties (including county-level cities) as rural and all districts as urban.

3. Urban areas: All the districts in the eastern, central and western regions are sorted according to the proportion of non-agricultural population and divided into three layers: high, medium and low. Then the urban areas of each layer are divided into three layers according to the total population, with a total of 27 layers.

4. Rural areas: All counties and county-level cities in the eastern, central and western regions are divided into 3 tiers according to the per capita GDP, and then each tier is divided into 3 tiers according to the total population, with a total of 27 tiers.

5. According to the national ratio of urban and rural areas, the ratio of urban and rural sample size is determined to be 2:3.

The steps for selecting monitoring sites from 2013 to 2019 are as follows:

1. According to the economic development status and population size of each province (autonomous region and municipality directly under the Central Government), determine the total number of 605 monitoring points in each province (autonomous region and municipality directly under the Central Government).

2. According to the results of the sixth national Population Census in 2010, all the districts and counties in each province (autonomous region, municipality directly under the Central Government) were stratified successively based on the three indicators of urbanization rate, population number and mortality rate. According to the three indicators, the districts and counties to be stratified were divided into two layers with the median as the dividing point, and the three indicators were divided into eight layers in total.

3. Determine the number of monitoring points at each layer based on the total number of monitoring points in each province (autonomous region, municipality directly under the Central Government).

4. Select selected districts and counties at different levels in each province (autonomous region, municipality directly under the Central Government), taking into account different geographical and population conditions.

5. The selected districts and counties in each province (autonomous region or municipality directly under the Central Government) shall be evaluated on provincial representatives. If the representatives is not good, the districts and counties in the same layer of the province (autonomous region or municipality directly under the Central Government) shall be replaced until all the selected districts and counties have provincial representatives; Finally determine the specific 605 monitoring points.
