# Supplementary material for: Trends in all-cause mortality and leading causes of death from 2009 to 2019 among older adults in China
Source: BMC Geriatr. 2023 Oct 11;23:645. doi: 10.1186/s12877-023-04346-7 (PMC10566094; doi:10.1186/s12877-023-04346-7)
Supplement: Supplementary file 2 — Supplementary Material 2 [file 12877_2023_4346_MOESM2_ESM.docx]

**Appendix 2**

Registration of deaths

Qualified medical practitioners and other specified medical and healthcare personnel can complete the Medical Certificate of Resident Death (the death certificate), including cause of death.

Deaths occurring in medical institutions (including death on arrival at hospital, death during pre-hospital emergency treatment, and death during hospital treatment) are required to be determined by the treating doctor, involving completion of a death certificate.

Where death occurs at home or in other locations, a local community doctor reports the death information to the respective township health centre (community health service centre), and doctors in the community health service centres deduce the cause of death and complete the death certificate based on the medical history, signs, and/or medical diagnosis provided by family members or others close to the deceased.

When a death requires the intervention of the public security judicial department, the department determines the cause of death and issues a death certificate. Doctors in community health service centres in the respective jurisdiction complete the death certificate accordingly.
