# Supplementary material for: Trends in all-cause mortality and leading causes of death from 2009 to 2019 among older adults in China
Source: BMC Geriatr. 2023 Oct 11;23:645. doi: 10.1186/s12877-023-04346-7 (PMC10566094; doi:10.1186/s12877-023-04346-7)
Supplement: Supplementary file 3 — Supplementary Material 3 [file 12877_2023_4346_MOESM3_ESM.docx]

**Appendix 3**

**Interview outline**

**Interview purpose**

Based on the findings of our study, we conducted a semi-structured expert interview on how to effectively implement health intervention among the elderly aged 65 and above in China. The interview was designed to provide scientific suggestions for the government to take effective health intervention measures and provide an evidence-based basis for improving the quality of life of the elderly and achieving healthy aging.

**Interview time**

July 1st to July 15th, 2022.

**Interview method**

Interviews were conducted online and offline, each interview lasted no less than 40 minutes and were conducted by two interviewers.

Prior to the interview, we explained the purpose of the interview to the interviewed experts and recorded the entire interview with the prior consent of the interviewed experts.

In the course of compiling information on the content of the interviews, we numbered the experts interviewed, standardized textual translation of the oral accounts of the experts interviewed on the basis of verbatim transcriptions, and removed oral language and meaningless repetitions from the interview narratives.

**Interview Content**

**1.Socio-demographic characteristics**

A1. What is your age?

A2. What is your nationality?

A3. What is your highest educational background?

A4. What is your title?

A5. What is your direction of research?

A6. What role do health promotion and intervention measures play in your daily work? Please describe an example.

**2.Trends of mortality among elderly in China**

B1. What do you think of the decline trend in mortality among older adults aged ≥65 in China from 2009 to 2019?

B2. What is your understanding of the higher mortality rate among males aged ≥65 than females in China and what interventions you think should be taken?

**3.Causes of death among elderly in China from 2009 to 2019**

C1. What is your understanding of the find that cerebrovascular disease was the leading cause of death among the older adults aged 65-84 years in China from 2009 to 2019 and what interventions do you think should be taken?

C2. What is your understanding of ischemic heart disease is the leading cause of death among the older adults aged ≥85 years in China, and what interventions do you think should be taken?

C3. What is your understanding of the find that falls accounted for the highest proportion of the injury causes of death among the older adults in China and shows an increasing trend, and what interventions do you think should be taken?
