# Supplementary material for: Trends in all-cause mortality and leading causes of death from 2009 to 2019 among older adults in China
Source: BMC Geriatr. 2023 Oct 11;23:645. doi: 10.1186/s12877-023-04346-7 (PMC10566094; doi:10.1186/s12877-023-04346-7)
Supplement: Supplementary file 4 — Supplementary Material 4 [file 12877_2023_4346_MOESM4_ESM.docx]

**Appendix 4** Cause of death rankings according to sex and age among older adults aged ≥65 years in China (2009-2019)

| **Years** | **65~69** | |  | **70~74** | |  | **75~79** | |  | **80~84** | |  | **≥85** | |
| --- | --- | --- | --- | --- | --- | --- | --- | --- | --- | --- | --- | --- | --- | --- |
|  | **Male** | **Female** |  | **Male** | **Female** |  | **Male** | **Female** |  | **Male** | **Female** |  | **Male** | **Female** |
| 2009 | CVD | CVD |  | CVD | CVD |  | CVD | CVD |  | CVD | CVD |  | CVD | CVD |
|  | IHD | IHD |  | COPD | IHD |  | COPD | IHD |  | COPD | COPD |  | COPD | IHD |
|  | Lung cancer | COPD |  | IHD | COPD |  | IHD | COPD |  | IHD | IHD |  | IHD | COPD |
| 2010 | CVD | CVD |  | CVD | CVD |  | CVD | CVD |  | CVD | CVD |  | CVD | CVD |
|  | IHD | IHD |  | COPD | IHD |  | COPD | IHD |  | COPD | IHD |  | IHD | IHD |
|  | Lung cancer | COPD |  | IHD | COPD |  | IHD | COPD |  | IHD | COPD |  | COPD | COPD |
| 2011 | CVD | CVD |  | CVD | CVD |  | CVD | CVD |  | CVD | CVD |  | CVD | CVD |
|  | IHD | IHD |  | IHD | IHD |  | IHD | IHD |  | COPD | IHD |  | IHD | IHD |
|  | Lung cancer | COPD |  | COPD | COPD |  | COPD | COPD |  | IHD | COPD |  | COPD | COPD |
| 2012 | CVD | CVD |  | CVD | CVD |  | CVD | CVD |  | CVD | CVD |  | CVD | CVD |
|  | IHD | IHD |  | IHD | IHD |  | IHD | IHD |  | COPD | IHD |  | IHD | IHD |
|  | Lung cancer | COPD |  | COPD | COPD |  | COPD | COPD |  | IHD | COPD |  | COPD | COPD |
| 2013 | CVD | CVD |  | CVD | CVD |  | CVD | CVD |  | CVD | CVD |  | CVD | CVD |
|  | IHD | IHD |  | IHD | IHD |  | IHD | IHD |  | IHD | IHD |  | IHD | IHD |
|  | Lung cancer | COPD |  | COPD | COPD |  | COPD | COPD |  | COPD | COPD |  | COPD | COPD |
| 2014 | CVD | CVD |  | CVD | CVD |  | CVD | CVD |  | CVD | CVD |  | CVD | IHD |
|  | IHD | IHD |  | IHD | IHD |  | IHD | IHD |  | IHD | IHD |  | IHD | CVD |
|  | Lung cancer | Lung cancer |  | COPD | COPD |  | COPD | COPD |  | COPD | COPD |  | COPD | COPD |
| 2015 | CVD | CVD |  | CVD | CVD |  | CVD | CVD |  | CVD | CVD |  | CVD | IHD |
|  | IHD | IHD |  | IHD | IHD |  | IHD | IHD |  | IHD | IHD |  | IHD | CVD |
|  | Lung cancer | Lung cancer |  | Lung cancer | COPD |  | COPD | COPD |  | COPD | COPD |  | COPD | COPD |
| 2016 | CVD | CVD |  | CVD | CVD |  | CVD | CVD |  | CVD | CVD |  | CVD | IHD |
|  | IHD | IHD |  | IHD | IHD |  | IHD | IHD |  | IHD | IHD |  | IHD | CVD |
|  | Lung cancer | Lung cancer |  | Lung cancer | COPD |  | COPD | COPD |  | COPD | COPD |  | COPD | COPD |
| 2017 | CVD | CVD |  | CVD | CVD |  | CVD | CVD |  | CVD | CVD |  | IHD | IHD |
|  | IHD | IHD |  | IHD | IHD |  | IHD | IHD |  | IHD | IHD |  | CVD | CVD |
|  | Lung cancer | Lung cancer |  | Lung cancer | COPD |  | COPD | COPD |  | COPD | COPD |  | COPD | COPD |
| 2018 | CVD | CVD |  | CVD | CVD |  | CVD | CVD |  | CVD | CVD |  | IHD | IHD |
|  | IHD | IHD |  | IHD | IHD |  | IHD | IHD |  | IHD | IHD |  | CVD | CVD |
|  | Lung cancer | Lung cancer |  | Lung cancer | COPD |  | COPD | COPD |  | COPD | COPD |  | COPD | COPD |
| 2019 | CVD | CVD |  | CVD | CVD |  | CVD | CVD |  | CVD | CVD |  | IHD | IHD |
|  | IHD | IHD |  | IHD | IHD |  | IHD | IHD |  | IHD | IHD |  | CVD | CVD |
|  | Lung cancer | Lung cancer |  | Lung cancer | Lung cancer |  | COPD | COPD |  | COPD | COPD |  | COPD | COPD |
